# Multiparametric CMR evaluation of myocardial remodeling in dystrophinopathy

**DOI:** 10.3389/fcvm.2026.1935273

**Published:** 2026-09-10

**Authors:** Wenting Luo, Jia Liu, Jie Wang, Yun Yuan, Jianxiu Lian, Wei Li, Shouyi Wei, Xiaofeng Qin, Wei Ma, Jianxing Qiu

**Affiliations:** 1 Department of Radiology, Peking University First Hospital, Beijing, China; 2 Department of Cardiology, Peking University First Hospital, Beijing, China; 3 School of Biological Science and Medical Engineering, Beihang University, Beijing, China; 4 Department of Radiology, Jincheng General Hospital, Jincheng, China

**Keywords:** cardiac magnetic resonance, dystrophinopathy, myocardial involvement, strain, t1 mapping

## Abstract

**Background:**

Cardiac involvement in dystrophinopathy progresses from diffuse myocardial remodeling to overt systolic failure. Multiparametric cardiac magnetic resonance (CMR) offers simultaneous insights into myocardial mechanics and tissue characterization, yet the optimal diagnostic parameters for tracking disease severity remain to be fully characterized.

**Objective:**

To objectively evaluate the descriptive and discriminative performance of integrating strain and quantitative T1 mapping parameters for identifying myocardial fibrosis burden and stratifying systolic dysfunction in patients with dystrophinopathy.

**Materials and methods:**

This retrospective study analyzed 55 patients with genetically confirmed dystrophinopathy and 30 healthy controls who underwent CMR between January 2020 and December 2024. Patients were sequentially stratified by late gadolinium enhancement (LGE) status (Group A: LGE-negative, *n* = 16; Group B: LGE-positive, *n* = 39), and further sub-stratified by left ventricular ejection fraction (LVEF) (Group C: LVEF ≥ 50%, *n* = 23; Group D: LVEF < 50%, *n* = 16). Least absolute shrinkage and selection operator (LASSO) regression with 10-fold cross-validation was applied for non-zero feature selection, followed by the construction of multivariable Firth penalized logistic regression models. Model comparisons were made using the non-parametric DeLong test.

**Results:**

Compared with Group A, Group B exhibited significant differences in LVEF (*p* < .001), mid-ventricular circumferential strain (CS_Mid, *p* < .001), and native T1 in segment 11 (T1map_seg11, mid-inferolateral, *p* = .004). In single-parameter ROC analysis, CS_Mid, LVEF, and T1map_seg11 exhibited an area under the curve (AUC) of 0.842 [95% confidence interval (CI): 0.735–0.949], 0.828 (95% CI: 0.714–0.942), and 0.715 (95% CI: 0.549–0.880), respectively. The combined model of CS_Mid and T1map_seg11 achieved the AUC of 0.888 (95% CI: 0.793–0.983). Compared Group C with Group D, CS_Global and T1map_seg5 models yielded AUCs of 0.921 (95% CI: 0.822–1.000) and 0.823 (95% CI: 0.694–0.953). The combined model (CS_Global + T1map_seg5) achieved the AUC of 0.951 (95% CI, 0.864–1.000, DeLong *p* = .567 vs T1map_seg5, DeLong *p* = .101 vs. T1map_seg5).

**Conclusions:**

In dystrophinopathy, multiparametric CMR enables structural and functional assessment of myocardial remodeling. Segmental native T1, particularly in the basal and mid-inferolateral segments, provides complementary information on regional myocardial tissue abnormalities. In contrast, CS accounts for most of the discrimination of disease severity. These findings suggest that the distinctive contribution of CMR may lie in regional myocardial tissue characterization rather than conventional functional assessment.

## 1 Introduction

Dystrophinopathy is an X-linked disorder caused by dystrophin deficiency, which destabilizes the sarcolemmal membrane and renders skeletal and cardiac myocytes vulnerable to contraction-induced injury, characterized by progressive skeletal myopathy and a developing cardiomyopathy phenotype (1, 2). It most commonly presents as Duchenne muscular dystrophy (DMD) and Becker muscular dystrophy (BMD), with an incidence of approximately 4.8 per 10,000 people and 1.6 per 100,000 people, respectively (3). With advancements in respiratory and multidisciplinary care extending survival, cardiac complications have emerged as a major driver of morbidity and long-term outcomes, shifting the therapeutic focus toward early detection and risk stratification of myocardial involvement (4).

Cardiomyopathy in dystrophinopathy progresses from early myocardial injury to fibrosis and overt systolic failure (5, 6). Conventional measures, such as clinical symptoms or left ventricular ejection fraction (LVEF), may miss these early stages of myocardial injury, which are often clinically silent (6, 7). Therefore, a sensitive imaging approach, cardiac magnetic resonance (CMR), is essential to guide risk stratification, timely cardioprotective therapy, delay heart failure, and improve prognosis.

CMR is a comprehensive, noninvasive, and multiparametric tool for identifying myocardial structure and function. CMR techniques, including late gadolinium enhancement (LGE), T1 mapping, extracellular volume (ECV) fraction quantification, and strain calculation, allow detection of early myocardial involvement, even when echocardiography and electrocardiogram appear normal (8). In dystrophinopathy, multiparametric CMR permits an integrated assessment of the potential pathologic trajectory, from diffuse interstitial changes to focal fibrosis and systolic impairment (9, 10). However, it remains uncertain whether imaging markers can reliably identify early dystrophinopathy or predict cardiomyopathy severity.

LGE primarily identifies focal replacement fibrosis. In DMD, multiple CMR studies have associated LGE presence with earlier progression of cardiomyopathy, supporting its role as a clinically meaningful imaging biomarker of established myocardial involvement (11). However, because LGE relies on regional gadolinium accumulation and consequent inversion recovery hyperenhancement within areas of expanded extracellular space, it may fail to detect globally or diffusely distributed interstitial expansion (12), limiting its sensitivity for earlier remodeling. T1 mapping, through assessment of native T1 and ECV, provides complementary, quantitative tissue characterization and has been demonstrated in DMD to detect diffuse interstitial abnormalities even in patients with preserved LVEF and no detectable LGE, highlighting its potential to detect pre-LGE remodeling (13, 14). However, whether these global tissue and mechanical parameters universally alter before localized replacement fibrosis, or how they behaviorally diverge across specific sub-cohorts stratified by LGE status, requires deeper empirical characterization.

CMR feature-tracking (CMR-FT) is a postprocessing technique that quantifies myocardial deformation by tracking intrinsic image features on standard cine CMR, avoiding additional scan sequences. It provides sensitive, quantitative measures of myocardial mechanics and has identified subclinical dysfunction across various cardiomyopathies (15–17). In dystrophinopathy, several studies have reported abnormal CMR-FT–derived strain despite preserved LVEF, indicating mechanical impairment that may precede conventional volumetric decline (18–20). However, it remains unclear whether strain abnormalities consistently precede focal replacement fibrosis detectable by LGE. One previous study has reported strain abnormalities even in patients without LGE (18), whereas others suggest that strain is not a replacement for LGE and may only be informative in a limited subset of patients with DMD (21, 22). Thus, it remains unclear which measures provide the most clinically relevant information and how tissue remodeling and mechanical dysfunction coevolve across disease stages, from fibrosis detection to overt systolic failure.

Therefore, this study aimed to assess cardiac tissue characteristics using CMR, considering fibrosis burden and functional status, with a distinct focus on stratification by myocardial fibrosis burden and systolic functional status. We hypothesized that myocardial deformation and tissue metrics would provide incremental and complementary diagnostic value for detecting early myocardial remodeling. Furthermore, we postulated that integrating strain analysis and segmental T1 mapping within a structured, multiparametric framework would provide a more comprehensive characterization to capture the cross-sectional severity of dystrophinopathy-related cardiomyopathy than conventional univariable indices.

## 2 Materials and methods

### 2.1 Study population

**Figure 1:**
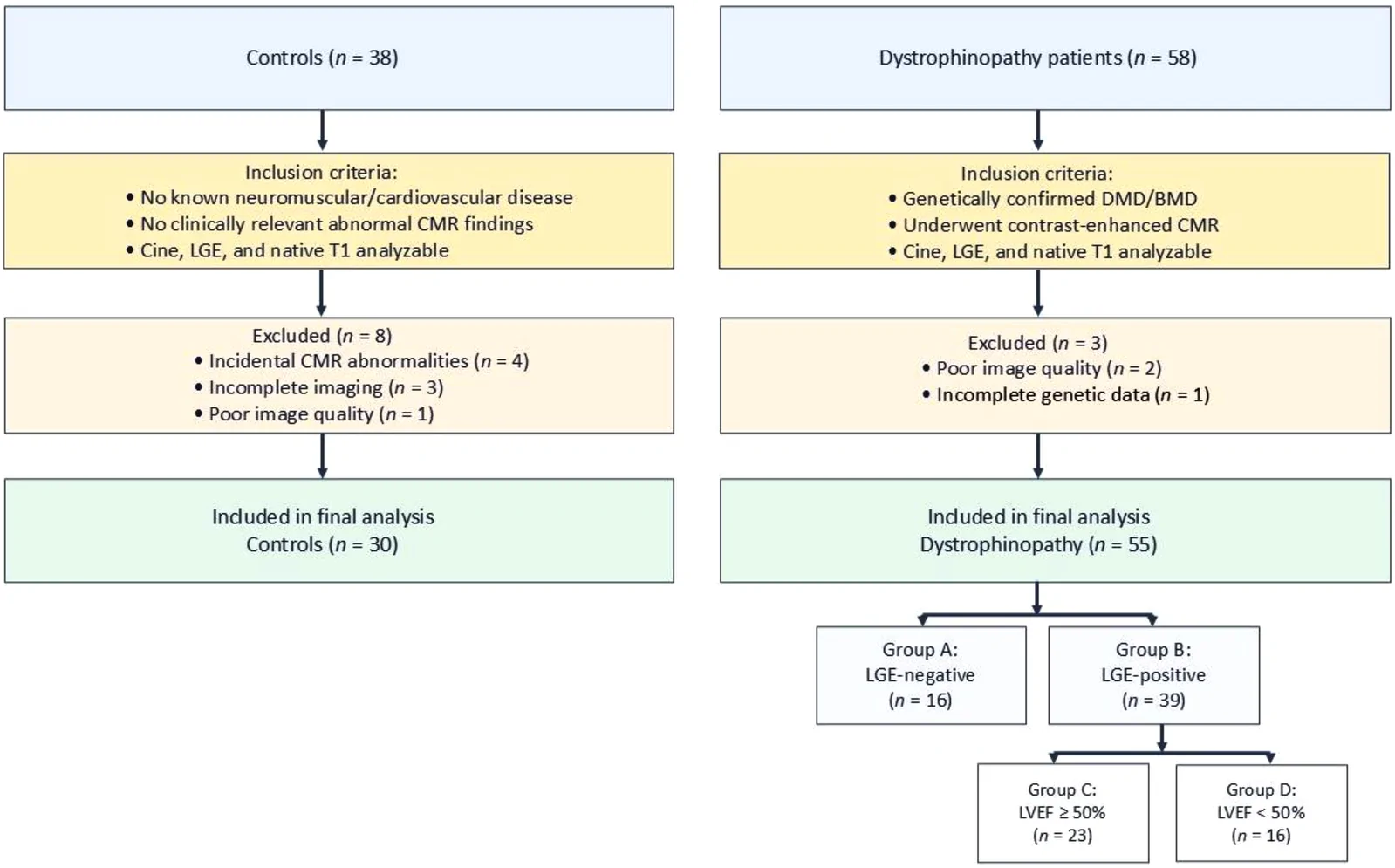
Study enrollment, flow diagram of participant selection, and subgroup stratification. The flow diagram summarizes screening, inclusion, exclusion, and final subgroup allocation of both controls and patients with dystrophinopathy. CMR, cardiac magnetic resonance; LGE, late gadolinium enhancement; LVEF, left ventricular ejection fraction.

This cross-sectional study consecutively included patients with genetically confirmed dystrophinopathy (BMD/DMD) who underwent clinically indicated CMR at our institution between January 2020 and December 2024. Patient inclusion criteria were as follows: (1) genetically confirmed DMD/BMD, (2) underwent contrast-enhanced CMR, and (3) analyzable cine, LGE, and native T1 images. The exclusion criteria were as follows: (1) standard contraindications to CMR, (2) prior myocardial infarction or other non-dystrophinopathy-related cardiomyopathy, and (3) incomplete datasets precluding quantification of ventricular function, LGE, strain, or T1 map (Figure 1). Controls inclusion criteria were as follows: (1) no known neuromuscular or cardiovascular disease, (2) no clinically relevant abnormal CMR findings, and (3) analyzable cine, LGE, and native T1 images. The exclusion criteria were as follows: (1) standard contraindications to CMR, and (2) incomplete datasets precluding quantification of ventricular function, LGE, strain, or T1 map (Figure 1). The study protocol was approved by the institutional review board, and informed consent was obtained. Patients were categorized *a priori* according to (1) LGE status: LGE-negative (Group A) versus LGE-positive (Group B), (2) systolic function: LVEF ≥50% according to the American Heart Association (AHA)'s ejection fraction heart failure measurement^24^ (Group C) versus LVEF <50% (Group D) (44).

### 2.2 CMR acquisition

All CMR examinations were performed on a 3.0-Tesla scanner (Ingenia; Philips Healthcare, Best, the Netherlands). Standard breath-hold cine images were acquired using balanced steady-state free precession sequences in long-axis and short-axis planes covering the left ventricle from base to apex. Native T1 mapping was performed in short-axis planes (basal, mid, and apical) using a modified Look-Locker inversion recovery (MOLLI) sequence with a 5s(3s)3s acquisition scheme with inline automated motion correction. Image quality control was visually performed by excluding images with severe breathing artifacts or susceptibility artifacts caused by adjacent lung gas. LGE images were obtained 7–10 min after intravenous administration of gadoterate meglumine (Dotarem, Guerbet, Ukraine) with the dose of 0.2 mmol/kg, using a phase-sensitive inversion recovery (PSIR) sequence in matching planes. Other protocols included black-blood short tau inversion recovery imaging and first-pass perfusion imaging. Detailed acquisition parameters are provided in Table 1.

**Table 1:** Sequence-specific cardiac magnetic resonance acquisition parameters and contrast administration protocols.

Sequence	Typical acquisition parameters						Contrast (gadoterate meglumine, Dotarem, Guerbet) administration protocol			
	TR (ms)	TE (ms)	Flip angle (°)	FOV (mm²)	Slice thickness (mm)	Slice gap (mm)	Dose (mmol/kg)	Injection rate (mL/s)	Saline injection rate (mL/s)	Acquisition
bSSFP	2.8	1.4	45	350 × 350	10	0	-			
Black-blood STIR	2,000	80	90	300 × 300	10	0	-			
MOLLI	2.1	1	20	300 × 300	10	-	-			
First-pass perfusion	2.5	1.18	15	300 × 300	10	10	0.1	2.0–3.0	2.0–3.0	Imaging started simultaneously with the injection
PSIR	6.1	3	25	350 × 350	10	0	0.2 (Additional)	1.0–2.0	1.0–2.0	Imaging 7–10 min after injection

Typical sequence-specific acquisition parameters and contrast administration settings are summarized. Parameter settings were adjusted as needed in individual examinations to account for patient heart rate, breath-hold capacity, and image quality. First-pass perfusion imaging was started simultaneously with contrast injection, whereas PSIR-LGE imaging was performed approximately 7–10 min after the additional contrast dose

bSSFP, balanced steady-state free precession; FOV, field of view; MOLLI, modified look-locker inversion recovery; PSIR, phase-sensitive inversion recovery; STIR, short tau inversion recovery; TE, echo time; TR, repetition time

### 2.3 Image analysis

Image analysis was performed using commercially available software (Cvi42; Circle Cardiovascular Imaging Inc., Calgary, Alberta, Canada). LVEF was quantified from short-axis cine stacks. Left ventricular end-diastolic volume (LVEDV), left ventricular end-systolic volume (LVESV), stroke volume (SV), and myocardial mass (MyoMass) were calculated according to standard conventions and indexed to body surface area (BSA).

CMR-FT strain analysis was performed on cine images to derive global and segmental radial strain (RS), circumferential strain (CS), and longitudinal strain (LS). Specifically, CS and LS are expressed as negative values, with less negative values indicating impaired systolic function; in contrast, RS is positive, with lower values indicating impaired systolic function. Endocardial and epicardial contours were defined at end-diastole and automatically propagated throughout the cardiac cycle, with manual adjustments as needed. Native T1 values were analyzed using the AHA 17-segment method. Regions of interest (ROIs) were conservatively drawn within the mid-myocardial layer of each segment, ensuring a margin from both endocardial and epicardial borders to avoid blood pool or fat partial volume effects. Notably, Segment 17 (the apex cap) was excluded from both T1 mapping and feature-tracking strain analysis due to its anatomical thinning and high susceptibility to partial volume artifacts, resulting in 16 evaluated segments. All analyses were performed by two experienced readers (J.L., associate chief physician with 8 years of CMR experience; X.F.Q., associate chief physician with 5 years of CMR experience) who were blinded to clinical group assignments.

### 2.4 Statistical analysis

All statistical analyses were performed using SPSS Statistics v27.0 (IBM, IL, USA) or R v4.5.1 (R Core Team). Continuous variables were assessed using the Shapiro–Wilk test and presented as mean ± standard deviation for normally distributed data or median (interquartile range) otherwise. Categorical variables are presented as n (%). Two-group comparisons were performed using the Student t test or the Mann–Whitney U test, as appropriate. Comparisons among 3 or more groups were conducted using the Kruskal–Wallis test for non-normally distributed data, followed by Bonferroni-adjusted *post hoc* pairwise comparisons.

#### 2.4.1 Reproducibility analysis

To evaluate interobserver and intraobserver reproducibility, a subset of 20 randomly selected participants was re-analyzed. Intraobserver variability was assessed by the primary reader after a 4-week interval, and interobserver variability was determined by a second independent radiologist. Intraobserver's intraclass correlation coefficients (ICCs) were calculated using a two-way mixed-effects model for absolute agreement with single-measure estimates. Interobserver's ICCs were calculated using a two-way random-effects model for absolute agreement based on single measurements. Ninety-five percent confidence intervals were estimated using paired bootstrap resampling with 1,000 iterations. ICC values were interpreted as poor (<0.50), moderate (0.50–0.75), good (0.75–0.90), or excellent (>0.90) agreement.

#### 2.4.2 Select variables

To robustly select diagnostic parameters from wide-ranging multi-parametric CMR metrics while minimizing the risk of overfitting inherent to our small sample size, we implemented a Least Absolute Shrinkage and Selection Operator (Lasso) multivariable logistic regression framework. To guarantee regularized generalization and minimize classification error, the Lasso hyperparameter (*λ*) tuning was optimized using a stratified 10-fold cross-validation framework based on minimum binomial deviance criteria. Especially, between Group C and D, chronological age was forced into the variable selection sequence as a mandatory covariate.

#### 2.4.3 Logistic regression models

The cross-validated imaging features were advanced into a multivariable Firth's penalized likelihood logistic regression model. The Firth correction incorporates a Jeffreys invariant prior into the score function, effectively penalizing the likelihood to output stable, finite, and unbiased regression coefficients (*β*), adjusted odds ratios (aORs), and robust standard errors.

#### 2.4.4 Model comparisons

The discriminative stability and classification accuracy of the configured multivariable equations were rigorously evaluated using Bootstrap internal validation with 1,000 resamples. This iterative resampling workflow rendered an optimism-corrected area under the curve (AUC) and its 95% confidence intervals (CIs). The optimal diagnostic cut-off coordinates were parameterized via the Youden index. Receiver operating characteristic (ROC) curves were constructed, and the statistical comparisons between independent or nested diagnostic models were conducted using the non-parametric DeLong test. A 2-sided *P* value <.05 was considered statistically significant. Significance levels in figures are denoted as *p < .05, **p < .01, and ***p < .001.

## 3 Results

### 3.1 Study population and stratification

A total of 96 participants (58 patients and 38 controls) were enrolled in this study. Of the 38 controls, 8 were excluded due to incidental CMR abnormalities, incomplete imaging, or poor image quality. Of the 58 patients with genetically confirmed dystrophinopathy, 3 were excluded due to poor image quality or incomplete genetic data (Figure 1). Ultimately, 55 patients with genetically confirmed dystrophinopathy and 30 healthy controls were included in this cross-sectional analysis.

Patients were categorized according to LGE status and systolic function. Specifically, patients with dystrophinopathy were initially stratified into an LGE-negative group (Group A, *n* = 16) and an LGE-positive group (Group B, *n* = 39). Group B was further subdivided based on LVEF into preserved systolic function (Group C: LVEF ≥ 50%, *n* = 23) and overt systolic dysfunction (Group D: LVEF < 50%, *n* = 16).

Reproducibility analysis demonstrated good to excellent intraobserver and interobserver agreement across all major evaluated parameters. Detailed ICC values and 95% confidence intervals are provided in Supplementary Table S1-2.

### 3.2 Global myocardial remodeling in dystrophinopathy

**Figure 2:**
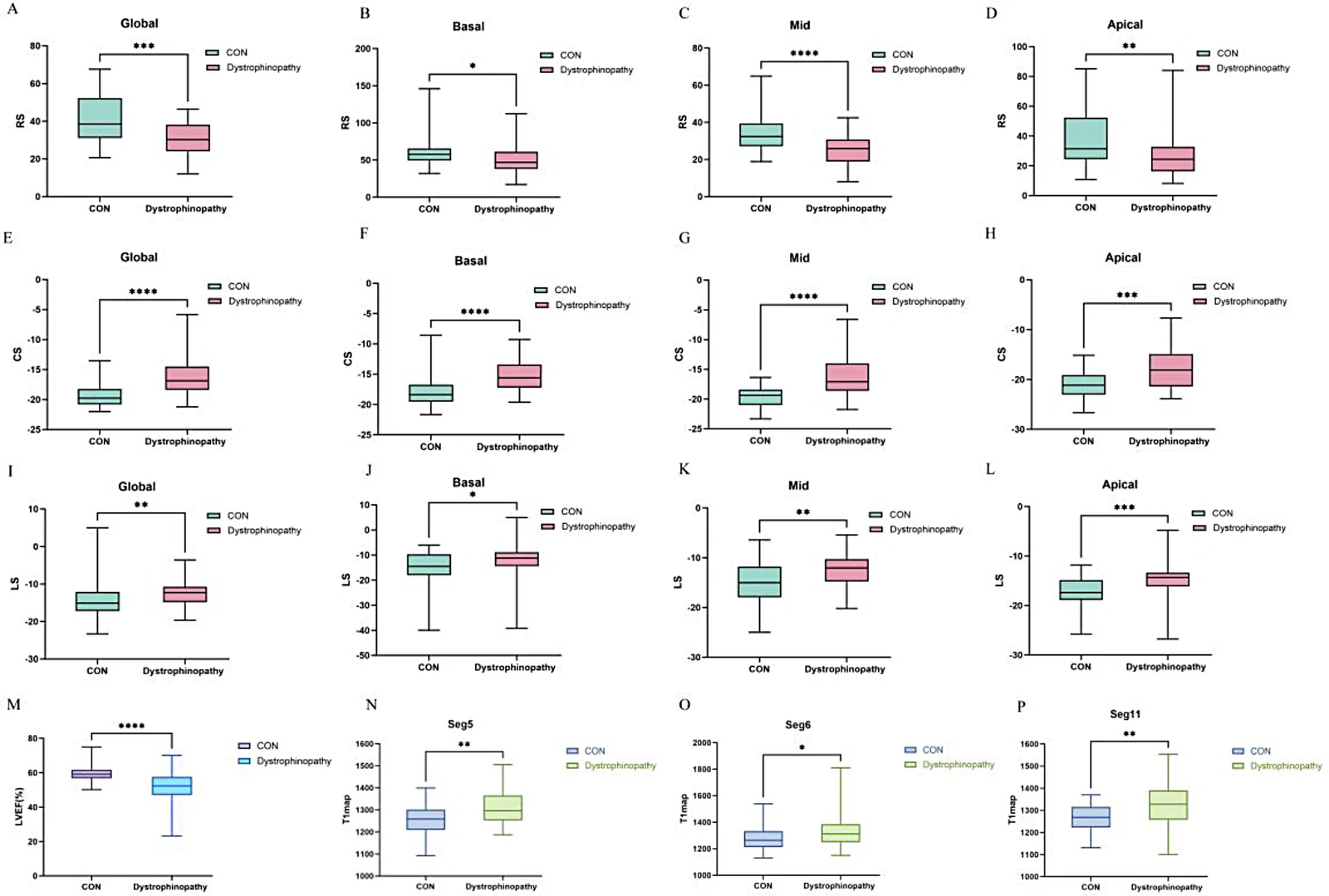
Abnormal CMR parameters in dystrophinopathy compared with controls. **(A–D)** RS at global, basal, mid, and apical levels; **(E–H)** CS at global, basal, mid, and apical levels; **(I–L)** LS at global, basal, mid, and apical levels; **(M)** LVEF; **(N–P)** native T1 values at segments 5, 6, and 11, corresponding to basal inferolateral, basal anterolateral, and mid-inferolateral segments, respectively. *p < .05, **p < .01, ***p < .001, ****p < .0001. CMR, cardiac magnetic resonance; Con, control; CS, circumferential strain; LS, longitudinal strain; LVEF, left ventricular ejection fraction; ns, not significant; RS, radial strain.

Global systolic function was impaired in patients, as reflected by a lower LVEF (*p* < .001) and increased LVESV/BSA (*p* = .006) (Table 2, Figure 2). Myocardial strain analysis demonstrated significant impairment in RS, CS, and LS at the basal (*p* = .016, <.001, and.008, respectively), mid (*p* < .001, <.001, and.003, respectively), apical (*p* = .003,.001, and <.001, respectively), and global (*p* < .001, <.001, and.010, respectively) levels (Supplementary Table S7, Supplementary Figure S1). For tissue characterization, native T1 values were higher in patients, with significant elevations in segment 5 (*p* = .003), 6 (*p* = .030), and 11 (*p* = .004), corresponding to the basal inferolateral, basal anterolateral, and mid-inferolateral segments, respectively (Figure 2).

**Table 2:** Baseline and CMR parameters in controls and dystrophinopathy.

Demographic profile	Controls	Dystrophinopathy	*P* value
Sample	*n* = 30	*n* = 55	
Age, year	14.42 ± 6.00	16.53 ± 8.46	.513
Male, *n*	16 (53.33%)	54 (98.18%)	<.001
Height, cm	156.43 ± 18.72	147.89 ± 18.66	.100
Weight, kg	53.5 (35.25–71.25)	48.85 ± 16.05	.358
LVEF, %	59.17 (56.83–61.64)	51.58 ± 10.05	<.001
LVEDV/BSA, mL/m^2^	95.56 ± 28.91	80.44 ± 21.64	.221
LVESV/BSA, mL/m^2^	29.18 ± 6.47	40.14 ± 18.05	.006
SV/BSA, mL/m^2^	43.05 ± 8.07	39.24 (33.69–47.77)	.232
MyoMass/BSA_diast, g/m^2^	41.1 ± 9.67	44.38 (36.55–51.57)	.051
MyoMass/BSA_syst, g/m^2^	43.14 ± 10.18	46.6 (38.24–52.8)	.132
Strain			
RS_Basal	68.3 ± 39.72	52.06 ± 22.12	.016
RS_Mid	35.16 ± 11.59	26.95 ± 17.38	<.001
RS_Apical	42.22 ± 30.51	27.53 ± 19.99	.003
RS_Global	38.49 (31.12–52.26)	31.09 ± 13.28	<.001
CS_Basal	−17.08 ± 5.38	−15.59 (−17.14, −13.36)	<.001
CS_Mid	−19.36 (−20.98, −18.42)	−16.11 ± 3.42	<.001
CS_Apical	−21.17 (−23.04, −19.12)	−17.53 ± 5.08	.001
CS_Global	−19.76 (−20.81, −18.25)	−16.17 ± 3.21	<.001
LS_Basal	−14.38 ± 7.65	−9.71 ± 8.57	.008
LS_Mid	−15.03 (−17.94, −11.8)	−12.05 (−14.53, −10.25)	.003
LS_Apical	−17.39 (−18.85, −14.87)	−14.75 ± 3.25	<.001
LS_Global	−14.31 ± 4.99	−12.59 ± 3.34	.010
T1 mapping			
T1map_seg1	1,280.55 (1,251.73–1,350.14)	1,336.4 ± 133.92	.392
T1map_seg2	1,295.38 (1,252.51–1,318.44)	1,306.45 (1,270.66–1,367.96)	.165
T1map_seg3	1,297.41 ± 87.51	1,288.67 (1,255.35–1,332.84)	.829
T1map_seg4	1,285.95 (1,233.25–1,316.45)	1,304.13 ± 104.94	.496
T1map_seg5	1,258.64 (1,209.6–1,301.11)	1,314.84 ± 80.3	.003
T1map_seg6	1,264.08 (1,213.66–1,331.49)	1,332.55 ± 117.12	.030
T1map_seg7	1,283.81 (1,246.26–1,379.31)	1,321.73 ± 98.1	.686
T1map_seg8	1,306.75 (1,257.9–1,330.42)	1,304.31 (1,261.89–1,338.03)	.520
T1map_seg9	1,272.89 (1,247.85–1,331.44)	1,293.71 (1,253.33–1,340.3)	.316
T1map_seg10	1,265.34 (1,225.32–1,317.79)	1,286.83 (1,244.25–1,341.52)	.225
T1map_seg11	1,268.4 (1,222.2–1,315.05)	1,327.98 (1,256.56–1,380.09)	.004
T1map_seg12	1,304.53 ± 74.5	1,367.63 ± 116.43	.307
T1map_seg13	1,355.36 ± 101.1	1,356.06 ± 97.24	.762
T1map_seg14	1,349.89 (1,253.66–1,438.75)	1,338.16 ± 100.63	.912
T1map_seg15	1,325.22 ± 110.88	1,366.19 ± 89.55	.485
T1map_seg16	1,360.74 (1,273.41–1,451.93)	1,367.63 ± 116.43	.949

Data are presented as mean ± SD for normally distributed variables and as median (interquartile range, IQR) for non-normally distributed variables. Categorical variables are presented as *n* (%). Between-group comparisons were performed using the Mann–Whitney *U* test for non-normally distributed continuous variables. Categorical variables were compared using Fisher's exact test. Segmental native T1 values correspond to the AHA 17-segment

AHA, American heart association; BSA, body surface area; LVEDV, left ventricular end-diastolic volume; LVEF, left ventricular ejection fraction; LVESV, left ventricular end-systolic volume; MyoMass, myocardial mass; RS/CS/LS, radial/circumferential/longitudinal strain; SD, standard deviation; SV, stroke volume; T1map, native T1 mapping (ms)

To rule out potential confounding effects of gender mismatches between the patients and the control group, we restrict the control group exclusively to male individuals (*n* = 16). RS (basal), CS (basal, mid, global, apical), and LS (mid), as well as the elevations in native T1 values (segments 2, 5, 6, 11, 16), showed statistically significant (all *p* < .05; detailed data are summarized in Supplementary Table S3).

### 3.3 Multiparametric CMR abnormalities stratified by myocardial fibrosis Status

Compared with Group A, Group B exhibited lower LVEF (*p* = .001) and higher LVESV/BSA (*p* < .001), increased indexed myocardial mass, and impaired strain, with a significant reduction in mid-ventricular circumferential strain (CS_Mid) [Group A: −19.24 (−20.31, 17.51) vs. Group B: −15.06 ± 3.39; *p* = .001]. Regional tissue abnormalities were also present: segmental native T1 values were also higher in Group B, particularly in segments 5 [Group A: 1233.77 (1223.82–1,280.21) vs. Group B: 1,332.52 ± 76.05; *p* = .012] and 11 [Group A: 1,248.37 (1,217.72–1,324.97) vs. Group B: 1,335.06 (1,290.15–1,402.46); *p* = .023], corresponding to basal inferolateral and mid-inferolateral segments (Table 3, Supplementary Table S8, Supplementary Figure S2).

**Table 3:** Baseline and CMR parameters across controls, group A, and group B.

Demographic profile	Controls	Group A	Group B
Sample	*n* = 30	*n* = 16	*n* = 39
Age, year	14.42 ± 6.00	12.31 ± 4.70	15.28 ± 6.3
Male, *n*	16 (53.33%)	15 (93.75%)*	39 (100%)*
LVEF, %	59.17 (56.83–61.64)	58.16 (57.17–63.78)	48.61 ± 10.01^*,#^
LVEDV/BSA, mL/m^2^	95.56 ± 128.91	65.23 (59.77–75.94)	85.49 ± 22.67^#^
LVESV/BSA, mL/m^2^	29.18 ± 6.47	26.46 (23.2–31.4)	45.06 ± 18.91^*,#^
SV/BSA, mL/m^2^	43.05 ± 8.07	40.52 (33.66–45.14)	40.44 ± 9.74
MyoMass/BSA_diast, g/m^2^	41.1 ± 9.67	36.2 (32.99–43.17)	46.95 (39.39–53.11)^*,#^
MyoMass/BSA_syst, g/m^2^	43.14 ± 10.18	38.51 (33.21–44.47)	48.98 (40.73–54.82)^*,#^
Strain			
RS_Basal	68.3 ± 39.72	60.14 ± 24.96	48.8 ± 19.96*
RS_Mid	35.16 ± 11.59	28.84 (25.75–34.07)	25.69 ± 20.25^*,#^
RS_Apical	42.22 ± 30.51	30.29 (22.32–39.74)	26.58 ± 22.58*
RS_Global	38.49 (31.12–52.26)	37.95 (30.89–40.85)	29.59 ± 15.13*
CS_Basal	−17.08 ± 5.38	−17.52 (−18.42, −15.59)	−15.35 (−16.75, −13.09)^*,#^
CS_Mid	−19.36 (−20.98, −18.42)	−19.24 (−20.31, −17.51)	−15.06 ± 3.39^*,#^
CS_Apical	−21.17 (−23.04, −19.12)	−21.39 (−22.54, −19.26)	−16.34 ± 5.45^*,#^
CS_Global	−19.76 (−20.81, −18.25)	−18.86 (−20.09, −17.45)	−15.23 ± 3.23^*,#^
LS_Basal	−14.38 ± 7.65	−11.10 ± 5.93	−9.14 ± 9.45*
LS_Mid	−15.03 (−17.94, −11.8)	−14.72 (−17.26, −11.56)	−10.66 (−14.30, −7.74)*
LS_Apical	−17.39 (−18.85, −14.87)	−15.81 (−17.27, −14.99)	−14.28 ± 3.45*
LS_Global	−14.31 ± 4.99	−13.72 (−16.06,−-12.29)	−11.97 ± 3.47*
T1 mapping			
T1map_seg5	1,258.64 (1,209.6–1,301.11)	1,233.77 (1,223.82–1,280.21)	1,332.52 ± 76.05^*,#^
T1map_seg6	1,264.08 (1,213.66–1,331.49)	1,251.21 (1,217.9–1,337.64)	1,345.99 ± 119.71*
T1map_seg11	1,268.4 (1,222.2–1,315.05)	1,248.37 (1,217.72–1,324.97)	1,335.06 (1,290.15–1,402.46)^*,#^

Data are presented as mean ± SD for normally distributed variables and as median (interquartile range, IQR) for non-normally distributed variables. Categorical variables are presented as *n* (%). Overall comparisons across the 3 groups were performed using the Kruskal–Wallis test for non-normally distributed continuous variables. *Post hoc* pairwise comparisons were adjusted using the Bonferroni method. Categorical variables were compared using Fisher's exact test. Segmental T1 values follow the AHA 17-segment. *p < .05 versus controls; ^#^*p* <.05 versus Group A (Bonferroni-adjusted)

AHA, American heart association; BSA, body surface area; LVEDV, left ventricular end-diastolic volume; LVEF, left ventricular ejection fraction; LVESV, left ventricular end-systolic volume; MyoMass, myocardial mass; RS/CS/LS, radial/circumferential/longitudinal strain; SD, standard deviation; SV, stroke volume; T1map, native T1 mapping (ms)

**Figure 3:**
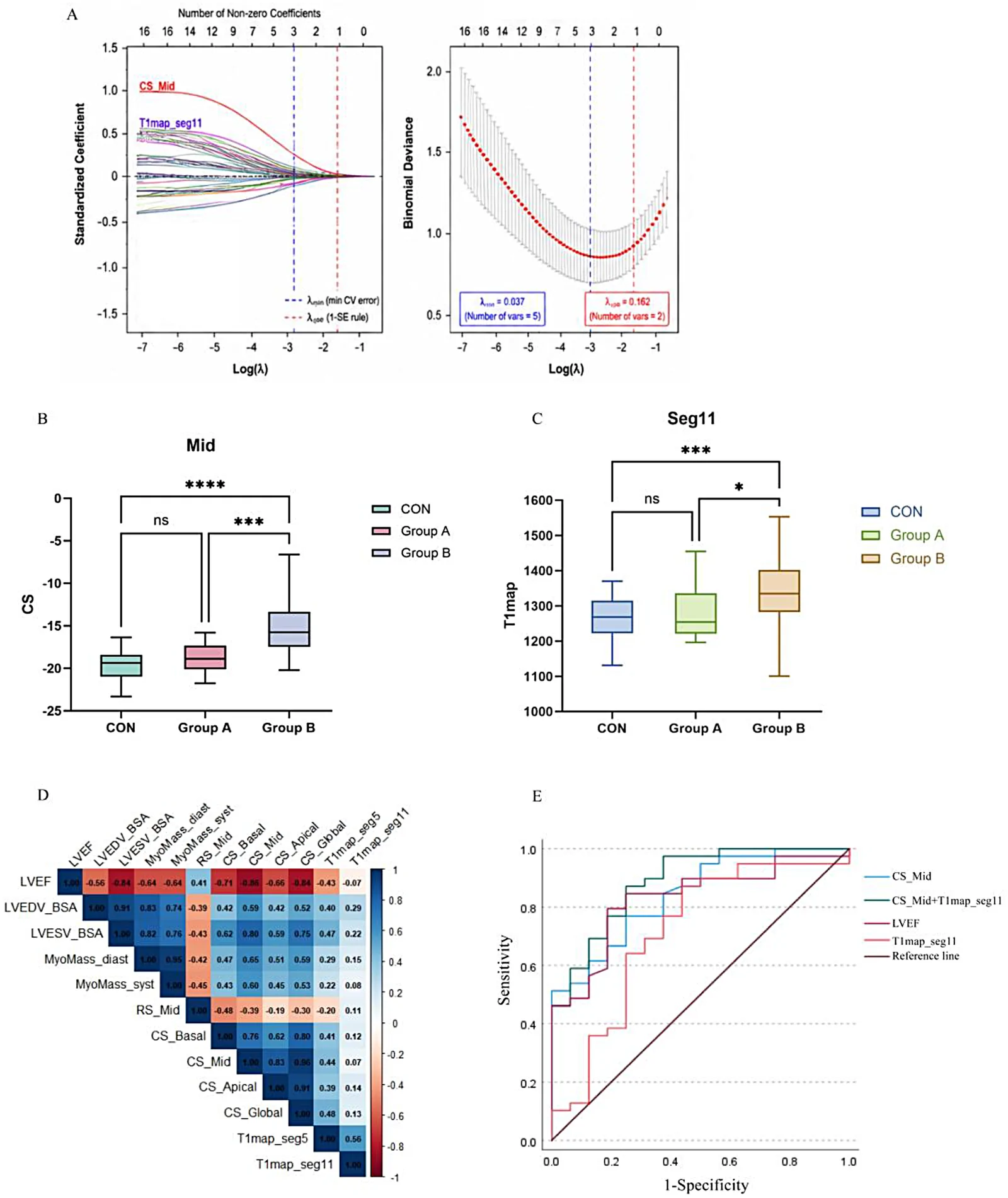
Major differential CMR parameters and discriminatory performance across controls, group A, and group B. **(A)** LASSO logistic regression with 10-fold cross-validation was performed. **(B)** CS at middle level, **(C)** native T1 value at segment 11 (corresponding to mid-inferolateral segment), **(D)** Spearman correlation heatmap among candidate CMR variables, and **(E)** ROC curves for discriminating Group B from Group A. *p < .05, **p < .01, ***p < .001, ****p < .0001. BSA, body surface area; CMR, cardiac magnetic resonance; Con, control; CS, circumferential strain; LS, longitudinal strain; LVEF, left ventricular ejection fraction; MyoMass, myocardial mass; ns, not significant; ROC, receiver operating characteristic; RS, radial strain.

CS_Mid and native T1 in segment 11 (T1map_seg11) were retained after feature selection (Figure 3), and their correlation was low (r = −0.07) (Figure 3). In ROC analysis for differentiating Group B from Group A, CS_Mid achieved an AUC of 0.842 (bootstrap 95% CI: 0.735–0.949; *p* < .001), whereas T1map_seg11 yielded an AUC of 0.715 (bootstrap 95% CI: 0.549–0.880; *p* = .011). LVEF demonstrated an AUC of 0.828 (bootstrap 95% CI: 0.714–0.942; *p* < .001). The combined model (CS_Mid + T1map_seg11) provided the highest AUC of 0.888 (bootstrap 95% CI: 0.793–0.983; *p* < .001) (Figure 3) and outperformed T1map_seg11 alone (DeLong test *p* = .019), but no significant difference with LVEF (DeLong test *p* > .05) (Tables 4 and Supplementary S4). The combined model demonstrated a sensitivity of 0.974 (95% CI: 0.852–0.997) and a specificity of 0.438 (95% CI: 0.224–0.677).

**Table 4:** ROC analysis for discriminating group A from group B and group C from group D.

Group A vs. Group B	AUC	Bootstrap 95% CI	*P* value
CS_Mid	0.842	0.735–0.949	<.001
CS_Mid + T1 map_seg11	0.888	0.793–0.983	<.001
LVEF	0.828	0.714–0.942	<.001
T1 map_seg11	0.715	0.549–0.880	.011
Group C vs. Group D			
Age	0.810	0.674–0.946	<.001
Age + CS_Global	0.921	0.822–1.000	<.001
Age + T1 map_seg5	0.823	0.694–0.953	<.001
Age + CS_Global + T1 map_seg5	0.951	0.864–1.000	<.001

ROC analysis was used to assess the discriminative performance of individual CMR parameters and their combined model. Data are presented as AUC, 95% CI, and *p* value

AUC, area under the curve; CI, confidence interval; CMR, cardiac magnetic resonance; CS, circumferential strain; LVEF, left ventricular ejection fraction; ROC, receiver operating characteristic

### 3.4 Functional differences with fibrotic phenotype and overt dysfunction

Within the LGE-positive cohort, patients in Group D were older than those in Group C and had significant differences in multiple myocardial deformation parameters. RS, CS, and LS were impaired across basal, mid, apical, and global levels. Notably, global circumferential strain (CS_Global) was −17.46 (−18.39, −16.18) in Group C versus −13.69 (−14.42, −9.6) in Group D (*p* = .007). Apical LS and T1map_seg5 [Group C: 1,287.43 (1,254.13–1,352.86), Group D: 1,362.77 (1,318.31–1,436.98); *p* = .023] also exhibited significant differences (Table 5).

**Table 5:** Baseline and CMR parameters across controls, group A, group C, and group D.

Demographic profile	Controls	Group A	Group C	Group D
Sample	*n* = 30	*n* = 16	*n* = 23	*n* = 16
Age, year	14.42 ± 6.00	12.31 ± 4.70	12.87 ± 5.84	18.75 ± 5.87^*,&^
Male, *n*	16 (53.33%)	15 (93.75%)*	23 (100%)*	16 (100%)*
Height, cm	156.43 ± 18.72	140.5 (134.25–150.75)	140 (130–150)	170 (146.25–177.25)^*,&^
Weight, kg	53.5 (35.25–71.25)	40.5 (35.75–50.25)	42 (36–52)	63.5 (46.25–74)^*,&^
LVEF, %	59.17 (56.83–61.64)	58.16 (57.17–63.78)	54.01 (51.3–57.49)	39.31 (36.38–46.05)^*,#,&^
LVEDV/BSA, mL/m^2^	95.56 ± 128.91	65.23 (59.77–75.94)	75.08 (62.26–93.71)	92.78 (77.12–117.08)^*,#^
LVESV/BSA, mL/m^2^	29.18 ± 6.47	26.46 (23.2–31.4)	34.43 (26.12–43.1)	57.84 (45.5–67.92)^*,#,&^
SV/BSA, mL/m^2^	43.05 ± 8.07	40.52 (33.66–45.14)	41.78 (34.35–50.61)	35.96 (27.95–46.3)^*,#^
MyoMass/BSA_diast, g/m^2^	41.1 ± 9.67	36.2 (32.99–43.17)	44.35 (37.88–50.47)	53.91 (46.63–57.72)^*,#^
MyoMass/BSA_syst, g/m^2^	43.14 ± 10.18	38.51 (33.21–44.47)	45.17 (39.47–52.6)	54.68 (48.19–60.41)^*,#^
RS_Basal	68.3 ± 39.72	60.14 ± 24.96	54.55 (46.53–73.79)	33.1 (21.19–44.99)^*,#,&^
RS_Mid	35.16 ± 11.59	28.84 (25.75–34.07)	27.03 (24.76–31.46)	15.36 (13.03–19.54)^*,#,&^
RS_Apical	42.22 ± 30.51	30.29 (22.32–39.74)	26.06 (17.75–40.54)	15.75 (11.14–19.92)^*,#,&^
RS_Global	38.49 (31.12–52.26)	37.95 (30.89–40.85)	34.04 (29.54–39.58)	18.2 (15.06–24.21)^*,#,&^
CS_Basal	−17.08 ± 5.38	−17.52 (−18.42, −15.59)	−16.19 (−17.09, −15.35)	−12.98 (−14.26, −10.77)^*,#,&^
CS_Mid	−19.36 (−20.98, −18.42)	−19.24 (−20.31, −17.51)	−17.28 (−18.21, −15.82)	−13.15 (−13.98, 9.62)^*,#,&^
CS_Apical	−21.17 (−23.04, −19.12)	−21.39 (−22.54, −19.26)	−19.47 (−21.4, −17.47)	−14.12 (−15.14, −10.6)^*,#,&^
CS_Global	−19.76 (−20.81, −18.25)	−18.86 (−20.09, −17.45)	−17.46 (−18.39, −16.18)	−13.69 (−14.42, −9.6)^*,#,&^
LS_Basal	−14.38 ± 7.65	−11.10 ± 5.93	−10.66 (−15.6, −7.59)	−9.8 (−12.72, −6.34)*
LS_Mid	−15.03 (−17.94, −11.8)	−14.72 (−17.26, −11.56)	−11.32 (−15.13, −10.5)	−10.59 (−13.23, −8.48)*
LS_Apical	−17.39 (−18.85, −14.87)	−15.81 (−17.27, −14.99)	−14.6 (−17.3, −13.99)	−12.73 (−13.97, −11.37)^*,#,&^
LS_Global	−14.31 ± 4.99	−13.72 (−16.06, −12.29)	−11.74 (−16.02, −10.44)	−11.34 (−12.72, −8.91)^*,#^
T1map_seg5	1,258.64 (1,209.6–1,301.11)	1,233.77 (1,223.82–1,280.21)	1,287.43 (1,254.13–1,352.86)	1,362.77 (1,318.31–1,436.98)^*,#,&^
T1map_seg11	1,268.4 (1,222.2–1,315.05)	1,248.37 (1,217.72–1,324.97)	1,327.98 (1,269.67–1,390.14)	1,343.69 (1,294.9–1,422.72)^*,#^

Data are presented as mean ± SD for normally distributed variables and as median (interquartile range, IQR) for non-normally distributed variables. Categorical variables are presented as *n* (%). Overall comparisons across the 4 groups were performed using the Kruskal–Wallis test for non-normally distributed continuous variables, with Bonferroni-adjusted *post hoc* pairwise comparisons. Categorical variables were compared using Fisher's exact test. *p < .05 versus controls; ^#^*p* <.05 versus Group A; ^&^*p* <.05 versus Group C, after Bonferroni adjustment

BSA, body surface area; LVEDV, left ventricular end-diastolic volume; LVEF, left ventricular ejection fraction; LVESV, left ventricular end-systolic volume; MyoMass, myocardial mass; RS/CS/LS, radial/circumferential/longitudinal strain; SD, standard deviation; SV: systolic volume; T1map: native T1 mapping (ms)

**Figure 4:**
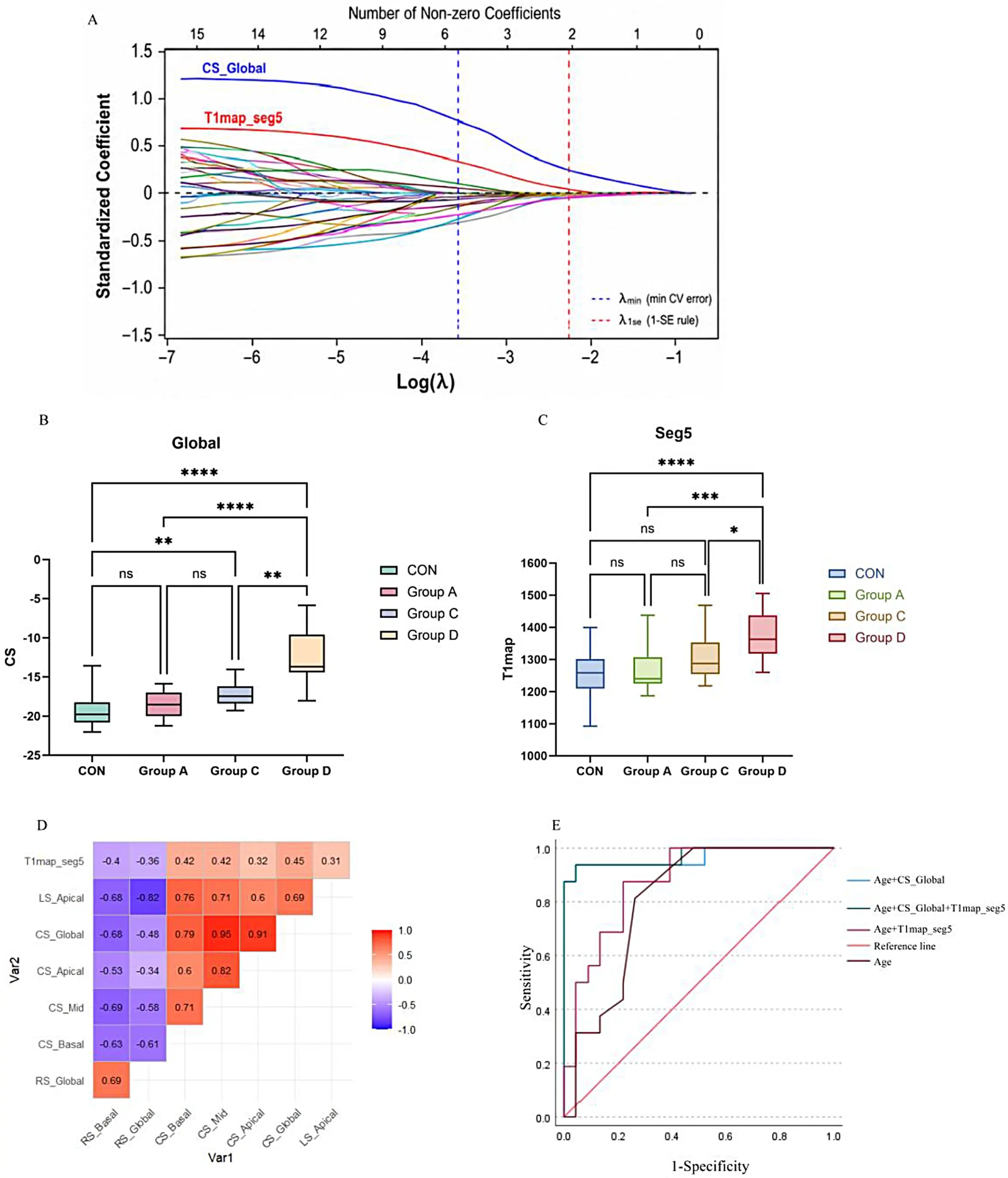
Major differential CMR parameters and discriminatory performance across controls, group C, and group D. **(A)** LASSO logistic regression with 10-fold cross-validation was performed. **(B)** CS at global level, **(C)** native T1 value at segment 5 (corresponding to basal inferolateral segment), **(D)** the heatmap displays pairwise Spearman correlation coefficients among main CMR variables, and **(E)** ROC curves based on predicted probabilities from the age-adjusted logistic model to discriminate Group C from Group D. *p < .05, **p < .01, ***p < .001, ****p < .0001. CMR, cardiac magnetic resonance; Con, control; CS, circumferential strain; LS, longitudinal strain; LVEF, left ventricular ejection fraction; MyoMass, myocardial mass; ns, not significant; ROC, receiver operating characteristic; RS, radial strain.

After adjustment for age, CS_Global (*β* = 1.564, *p* = .012) and T1map_seg5 (*β* = 0.024, *p* = .080) were retained in the final model (Figure 4, Supplementary Table S5). Correlation analysis demonstrated strong interdependence among strain-derived variables across RS, CS, and LS domains (Figure 4), whereas T1map_seg5 demonstrated modest correlations with strain (r = 0.45). The CS_Global and T1map_seg5 model yielded the AUC of 0.921 (bootstrap 95% CI: 0.822–1.000) and 0.823 (bootstrap 95% CI: 0.694–0.953). Adding T1map_seg5 to the age-adjusted CS_Global model increased the AUC to 0.951 (bootstrap 95% CI: 0.864–1.000; *Δ*AUC = 0.030) (Supplementary Table S6). The optimal probability cut-off determined by the Youden index was 0.067, and the sensitivity and specificity of the combined model were 0.938 (95% CI: 0.765–0.986) and 0.522 (95% CI: 0.298–0.738), respectively.

## 4 Discussion

**Figure 5:**
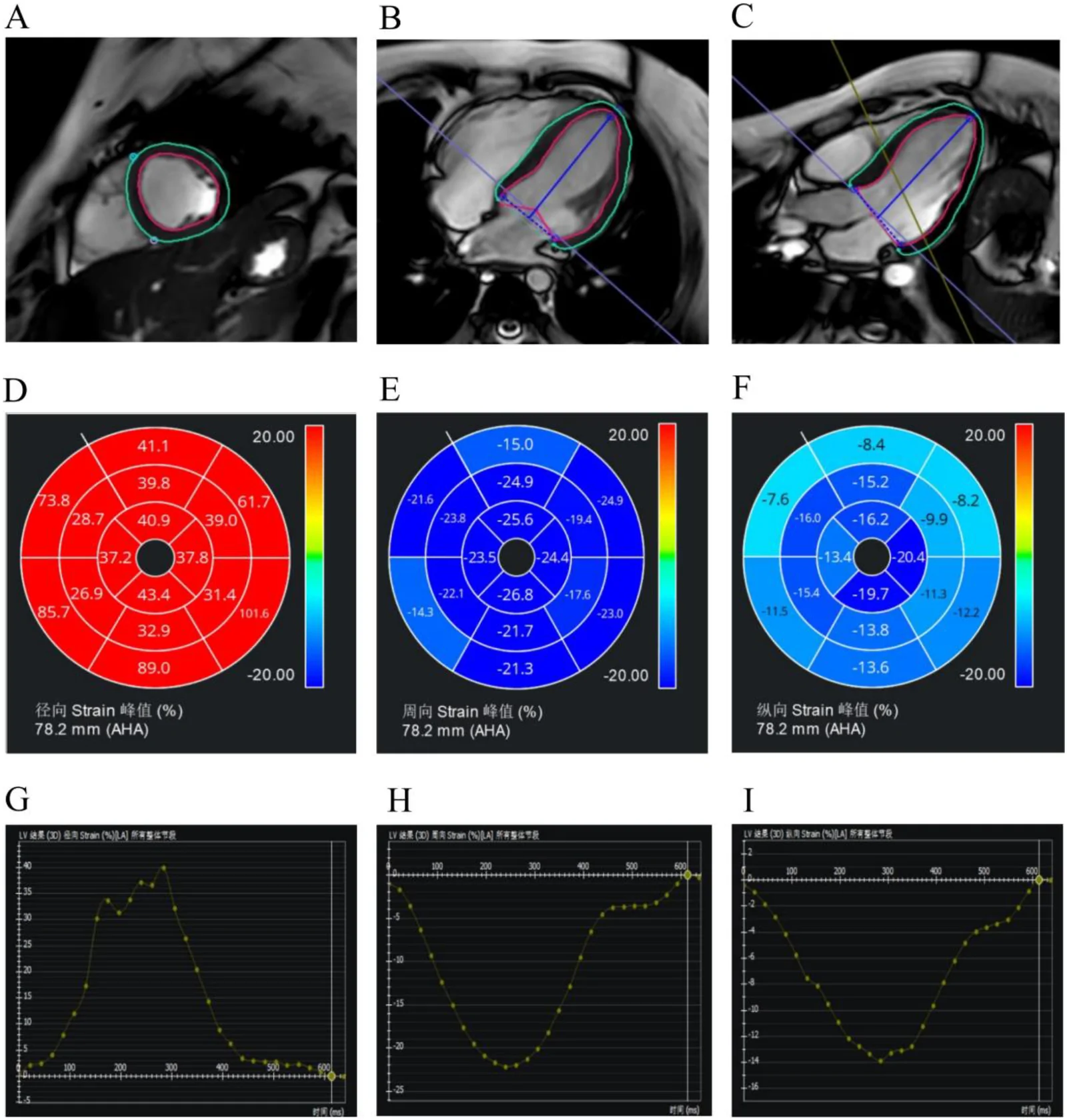
Representative CMR images and myocardial strain analysis in an 11-year-old male patient with dystrophinopathy and negative late gadolinium enhancement (LGE). **(A–C)** Cine images in the left ventricular short-axis, four-chamber, and three-chamber views, respectively. **(D–F)** Sixteen-segment bull's-eye plots of radial strain (RS), circumferential strain (CS), and longitudinal strain (LS), respectively. **(G–I)** Corresponding myocardial strain curves for RS, CS, and LS, respectively.

**Figure 6:**
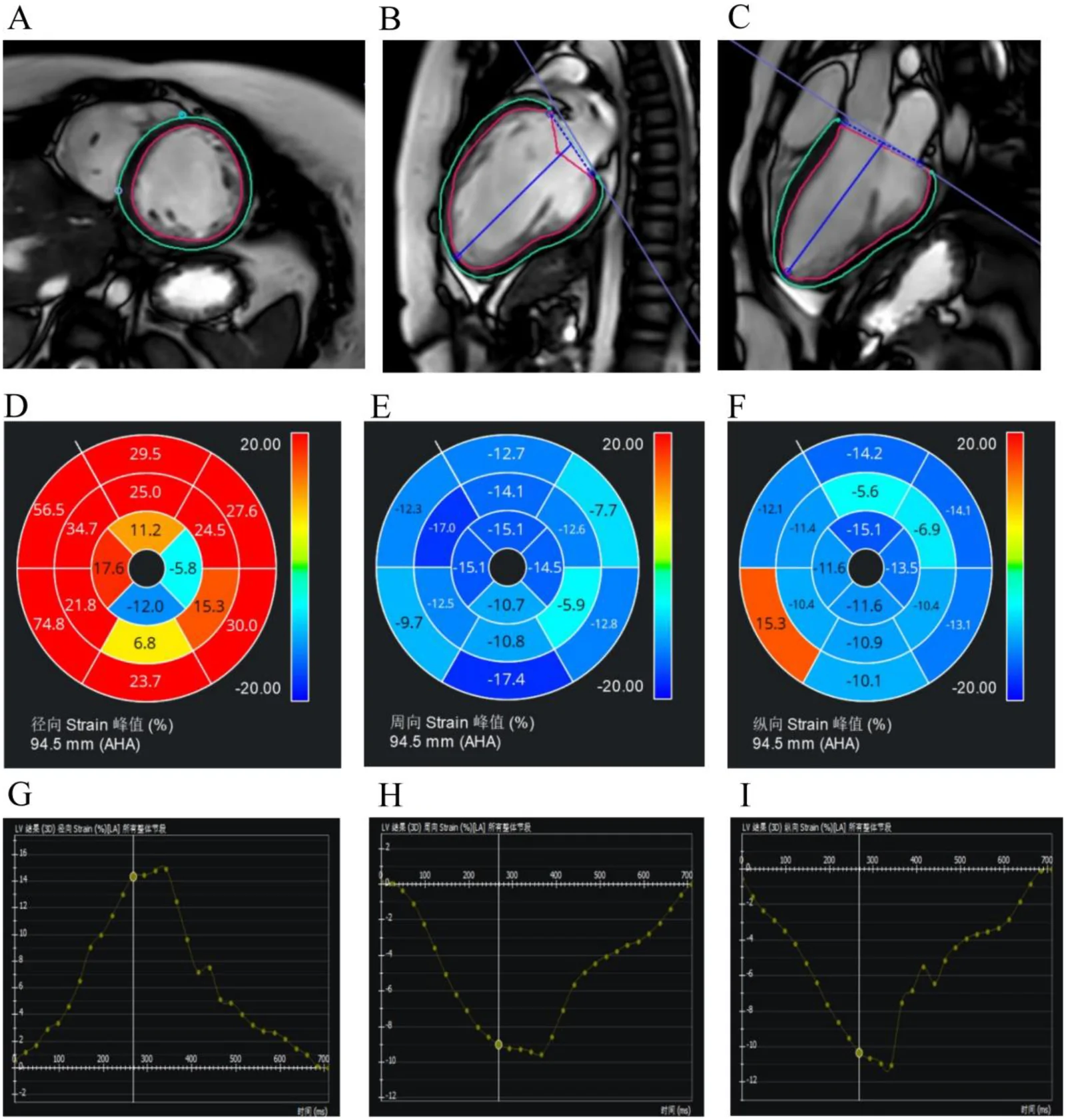
Representative CMR images and myocardial strain analysis in a 15-year-old male patient with dystrophinopathy and positive late gadolinium enhancement (LGE). **(A–C)** Cine images in the left ventricular short-axis, two-chamber, and three-chamber views, respectively. **(D–F)** Sixteen-segment bull's-eye plots of radial strain (RS), circumferential strain (CS), and longitudinal strain (LS), respectively. **(G–I)** Corresponding myocardial strain curves for RS, CS, and LS, respectively.

This study integrated myocardial tissue characterization and deformation analysis to clarify cardiac involvement in dystrophinopathy. The principal finding of this study is that the functional and tissue-characterization components of multiparametric CMR contributed differently to cross-sectional myocardial phenotyping in dystrophinopathy. Circumferential strain showed stronger discrimination between the LGE- and LVEF-defined phenotypes than segmental native T1, whereas native T1 abnormalities were regionally concentrated in the inferolateral myocardium. Adding regional native T1 to circumferential-strain models increased the AUC by only 0.046 for LGE status and 0.030 for the reduced-LVEF phenotype. Thus, our data do not show that quantitative native T1 is superior to functional assessment; rather, its main contribution appears to be complementary, spatially localized tissue information. Representative CMR images and myocardial strain analyses of LGE-negative and LGE-positive patients are shown in Figures 5, 6, respectively.

The observation that strain abnormalities emerge from LGE-negative status with preserved LVEF to reduced LVEF aligns with previous studies suggesting that strain can detect subclinical myocardial dysfunction before overt decline in LVEF (7, 21, 23, 24). Strain has been widely discussed as a sensitive functional biomarker in dystrophinopathy, particularly during the “silent” phase when symptoms may be masked by limited mobility and echocardiographic assessment is suboptimal (25). A central contribution of this study is the finding that, among intercorrelated strain parameters, mid-ventricular CS may be a valuable parameter for distinguishing LGE-positive patients with preserved versus reduced LVEF. This observation is consistent with established cardiac physiology, in which circumferential shortening plays a dominant role in left ventricular pump performance and becomes a major determinant of ejection fraction once compensatory mechanisms are exhausted (26). Conceptually, early disease may allow partial compensation through altered torsion, remodeling, or regional recruitment. However, beyond a threshold of circumferential mechanical failure, maintenance of global volumetric output becomes increasingly difficult, potentially representing an inflection point in disease progression (27, 28). The middle segment often represents a mechanically and geometrically pivotal region where circumferential shortening substantially contributes to cavity size reduction (29, 30). Although segmental vulnerability may vary depending on cohort characteristics and methodology, previous CMR studies have consistently highlighted the value of CS for staging and monitoring dystrophin-related cardiomyopathy (25). Our data extend this concept by demonstrating not only that CS is abnormal but also that mid-ventricular CS provides functional discrimination once fibrosis is present among other strains. This supports its potential role as a pragmatic marker of functional transition in clinical CMR workflows.

Notably, our results also support that native T1, which reflects diffuse interstitial remodeling, can provide additional information before marked impairment in cardiac function. This is consistent with previous studies on CMR demonstrating that T1 abnormalities can precede overt systolic dysfunction in dystrophin-related cardiomyopathy (31). This complementary relationship is also biologically plausible. Early cardiac involvement in dystrophin-related cardiomyopathy is more likely characterized by diffuse interstitial remodeling with regional and layer-specific heterogeneity, rather than widespread replacement fibrosis (32, 33). Furthermore, recent studies suggest that dystrophin-related cardiomyopathy involves complex fibrofatty and spatially heterogeneous remodeling, rather than fibrosis alone (34). Such microstructural remodeling may contribute to detectable strain aberrations before the development of overt volumetric dysfunction by impairing regional contractile synchrony and cardiac force transmission (35, 36). Elevated native T1 is a quantitative signal of tissue composition changes, encompassing extracellular matrix expansion, pre-focal diffuse fibrosis, and inflammatory alterations (37). In DMD-related cardiomyopathy, regional T1 heterogeneity may precede abnormalities detected by global average measurements (12).

Interestingly, our findings in Group C (LGE-positive with preserved LVEF) present a nuanced scenario that outer-shell contrasts with some prior investigations. While previous literature highlighted the potential of native T1 and strain to detect pre-LGE diffuse remodeling in dystrophinopathy (13, 14, 38), patients in our Group C exhibited global T1 and strain parameters that were statistically indistinguishable from healthy controls, despite already harboring positive LGE. This apparent discrepancy can be reconciled through the pathophysiological lens of spatial heterogeneity versus global averaging. In the early stages of dystrophinopathy, myocardial involvement is notoriously segmental, classically debuting in the epicardial or basal inferolateral walls (12, 39). LGE, evaluated as a high-contrast visual arbiter, possesses exquisite sensitivity for identifying these highly localized, dense patches of focal replacement fibrosis, thus turning positive early (39). Consequently, these global metrics remained within the statistically normal range during this initial focal stage. This finding does not invalidate the early diagnostic potential of T1 and strain reported previously, but rather underscores that in our cohort, localized replacement manifests with higher cross-sectional conspicuousness than generalized, global tissue or mechanical decay.

Our multivariate diagnostic models demonstrate a pronounced sensitivity-specificity trade-off. The sensitivity of the combined model is offset by compromised specificities. Subclinical myocardial remodeling in dystrophin-related cardiomyopathy progresses insidiously, frequently masked by restricted physical mobility (40). Under this clinical scenario, a false-negative classification carries catastrophic long-term consequences by delaying life-prolonging, guideline-directed cardioprotective therapies (41). Contemporary international consensus statements increasingly advocate for the early, often prophylactic introduction of safe, cost-effective medications, such as angiotensin-converting enzyme inhibitors or angiotensin receptor blockers, as these patients age, even before overt structural or systolic decay (42, 43). Rather than serving as standalone diagnostic gatekeepers, these parameters should be conceptualized as complementary alert mechanisms within a multi-parametric CMR workflow to prompt timely clinical surveillance or early therapeutic positioning.

This study had several limitations. First, due to the retrospective nature and the relatively constrained convenience sample size of this single-center registry, we were unable to perform high-level clinical decision-support evaluations, such as a Decision Curve Analysis (DCA), or to compute the Net Reclassification Improvement (NRI) and Integrated Discrimination Improvement (IDI) indices. Consequently, the net clinical benefit and exact reclassification efficacy of incorporating these advanced deformation and tissue parameters over conventional imaging frameworks require further validation in larger, multi-center prospective longitudinal cohorts. Second, dichotomizing systolic function using an LVEF threshold of 50% may oversimplify the severity of dystrophinopathy-related cardiomyopathy. However, this cutoff was selected for clinical interpretability, and future studies should further stratify LVEF to explore the impact of CMR strain and T1 mapping across disease stages. Third, this study is subject to the inherent limitations of a cross-sectional and retrospective design. Grouping patients by LGE status and LVEF allowed us to observe associations across different severity stages, but this cannot substitute for true longitudinal surveillance.

## 5 Conclusions

Myocardial circumferential strain provides valuable functional discrimination of severity, whereas segmental native T1, particularly in the basal inferolateral and mid-inferolateral segments, offers complementary information by identifying regional myocardial tissue abnormalities. Overall, these findings suggest that the distinctive contribution of CMR in dystrophinopathy may lie in regional myocardial tissue characterization and localization of injury beyond conventional functional assessment.

## Data Availability

The original contributions presented in the study are included in the article/Supplementary Material, further inquiries can be directed to the corresponding authors.
