# Supplementary material for: Multiparametric CMR evaluation of myocardial remodeling in dystrophinopathy
Source: Front Cardiovasc Med. 2026 Sep 10;13:1935273. doi: 10.3389/fcvm.2026.1935273 (PMC13601352; doi:10.3389/fcvm.2026.1935273)
Supplement: Supplementary file 1 [file Datasheet1.docx]

**Supplementary Materials Appendix**

**Contents**

[Table S1. Intraobserver reproducibility of major CMR parameters	2](#_Toc233371712)

[Table S2. Interobserver reproducibility of major CMR parameters	4](#_Toc233371713)

[Table S3. Gender-adjusted baseline and CMR parameters in controls and dystrophinopathy	6](#_Toc233371714)

[Table S4. Pairwise comparison of AUCs for discrimination of Group A versus Group B (DeLong test).	8](#_Toc233371715)

[Table S6. Pairwise comparison of AUCs for age-adjusted for discrimination of Group C versus Group D (DeLong test).	10](#_Toc233371716)

[Table S7. Non-significant baseline and CMR parameters in controls, Group A, and Group B	11](#_Toc233371717)

[Table S8. Non-significant CMR parameters in controls, GroupA, Group C, and Group D.	13](#_Toc233371718)

[Fig.S1. Major differential CMR parameters across controls, Group A, and Group B.	15](#_Toc233371719)

[Fig.S2. Major differential CMR parameters across controls, Group C, and Group D.	16](#_Toc233371720)

# Table S1. Intraobserver reproducibility of major CMR parameters

| **Parameters** | **N pairs** | **ICC(A,1)** | **Bootstrap 95% CI** | **p value** | **Interpretation** |
| --- | --- | --- | --- | --- | --- |
| LVEF | 20 | 0.993 | 0.981-0.997 | <0.001 | Excellent |
| LVEDV/BSA | 20 | 0.999 | 0.998-0.999 | <0.001 | Excellent |
| LVESV/BSA | 20 | 0.997 | 0.991-0.999 | <0.001 | Excellent |
| SV/BSA | 20 | 0.990 | 0.982-0.994 | <0.001 | Excellent |
| MyoMass/BSA_diast | 20 | 0.988 | 0.977-0.993 | <0.001 | Excellent |
| MyoMass/BSA_syst | 20 | 0.990 | 0.974-0.995 | <0.001 | Excellent |
| RS_Basal | 20 | 0.897 | 0.992-0.999 | <0.001 | Good |
| RS_Mid | 20 | 0.914 | 0.948-0.983 | <0.001 | Excellent |
| RS_Apical | 20 | 0.896 | 0.814-0.998 | <0.001 | Good |
| RS_Global | 20 | 0.923 | 0.782-0.991 | <0.001 | Excellent |
| CS_Basal | 20 | 0.844 | 0.643-0.910 | <0.001 | Good |
| CS_Mid | 20 | 0.828 | 0.706-0.959 | <0.001 | Good |
| CS_Apical | 20 | 0.907 | 0.895-0.968 | <0.001 | Excellent |
| CS_Global | 20 | 0.928 | 0.886-0.982 | <0.001 | Excellent |
| LS_Basal | 20 | 0.871 | 0.714-0.987 | <0.001 | Good |
| LS_Mid | 20 | 0.884 | 0.764-0.936 | <0.001 | Good |
| LS_Apical | 20 | 0.909 | 0.655-0.966 | <0.001 | Excellent |
| LS_Global | 20 | 0.898 | 0.756-0.945 | <0.001 | Good |
| T1map_seg1 | 20 | 0.899 | 0.796-1.000 | <0.001 | Good |
| T1map_seg2 | 20 | 0.806 | 0.781-0.998 | <0.001 | Good |
| T1map_seg3 | 20 | 0.904 | 0.819-0.998 | <0.001 | Excellent |
| T1map_seg4 | 20 | 0.909 | 0.814-1.000 | <0.001 | Excellent |
| T1map_seg5 | 20 | 0.917 | 0.794-0.998 | <0.001 | Excellent |
| T1map_seg6 | 20 | 0.899 | 0.797-1.000 | <0.001 | Good |
| T1map_seg7 | 20 | 0.918 | 0.826-0.999 | <0.001 | Excellent |
| T1map_seg8 | 20 | 0.836 | 0.760-0.898 | <0.001 | Good |
| T1map_seg9 | 20 | 0.897 | 0.793-0.998 | <0.001 | Good |
| T1map_seg10 | 20 | 0.818 | 0.795-0.899 | <0.001 | Good |
| T1map_seg11 | 20 | 0.808 | 0.715-0.909 | <0.001 | Good |
| T1map_seg12 | 20 | 0.817 | 0.787-0.939 | <0.001 | Good |
| T1map_seg13 | 20 | 0.819 | 0.714-0.969 | <0.001 | Good |
| T1map_seg14 | 20 | 0.831 | 0.715-0.939 | <0.001 | Good |
| T1map_seg15 | 20 | 0.901 | 0.831-1.000 | <0.001 | Excellent |
| T1map_seg16 | 20 | 0.827 | 0.714-0.998 | <0.001 | Good |

# Table S2. Interobserver reproducibility of major CMR parameters

| **Parameters** | **ICC(A,1)** | **Bootstrap 95% CI** | **p value** | **Interpretation** |
| --- | --- | --- | --- | --- |
| LVEF | 0.910 | 0.875–0.995 | <0.001 | Excellent |
| LVEDV/BSA | 0.938 | 0.812–0.999 | <0.001 | Excellent |
| LVESV/BSA | 0.908 | 0.723–0.979 | <0.001 | Excellent |
| SV/BSA | 0.913 | 0.818–0.996 | <0.001 | Excellent |
| MyoMass/BSA_diast | 0.938 | 0.878–0.992 | <0.001 | Excellent |
| MyoMass/BSA_syst | 0.899 | 0.771–0.994 | <0.001 | Good |
| RS_Basal | 0.898 | 0.795–0.973 | <0.001 | Good |
| RS_Mid | 0.874 | 0.750–0.984 | <0.001 | Good |
| RS_Apical | 0.906 | 0.819–0.976 | <0.001 | Excellent |
| RS_Global | 0.918 | 0.775–0.993 | <0.001 | Excellent |
| CS_Basal | 0.837 | 0.682–0.907 | <0.001 | Good |
| CS_Mid | 0.938 | 0.855–0.966 | <0.001 | Excellent |
| CS_Apical | 0.929 | 0.727–0.972 | <0.001 | Excellent |
| CS_Global | 0.935 | 0.841–0.959 | <0.001 | Excellent |
| LS_Basal | 0.864 | 0.693–0.978 | <0.001 | Good |
| LS_Mid | 0.843 | 0.735–0.891 | <0.001 | Good |
| LS_Apical | 0.880 | 0.569–0.946 | <0.001 | Good |
| LS_Global | 0.869 | 0.655–0.940 | <0.001 | Good |
| T1map_seg1 | 0.823 | 0.719–1.000 | <0.001 | Good |
| T1map_seg2 | 0.898 | 0.692–0.997 | <0.001 | Good |
| T1map_seg3 | 0.896 | 0.788–0.998 | <0.001 | Good |
| T1map_seg4 | 0.901 | 0.797–1.000 | <0.001 | Excellent |
| T1map_seg5 | 0.909 | 0.818–0.994 | <0.001 | Excellent |
| T1map_seg6 | 0.876 | 0.678–1.000 | <0.001 | Good |
| T1map_seg7 | 0.829 | 0.718–1.000 | <0.001 | Good |
| T1map_seg8 | 0.818 | 0.696–0.979 | <0.001 | Good |
| T1map_seg9 | 0.908 | 0.795–0.917 | <0.001 | Excellent |
| T1map_seg10 | 0.914 | 0.817–1.000 | <0.001 | Excellent |
| T1map_seg11 | 0.872 | 0.738–0.994 | <0.001 | Good |
| T1map_seg12 | 0.829 | 0.798–0.916 | <0.001 | Good |
| T1map_seg13 | 0.916 | 0.696–1.000 | <0.001 | Excellent |
| T1map_seg14 | 0.905 | 0.732–1.000 | <0.001 | Excellent |
| T1map_seg15 | 0.876 | 0.787–0.929 | <0.001 | Good |
| T1map_seg16 | 0.823 | 0.697–0.974 | <0.001 | Good |

# Table S3. Gender-adjusted baseline and CMR parameters in controls and dystrophinopathy

| **Demographic profile** | **Controls** | **Dystrophinopathy** | ***p* value** |
| --- | --- | --- | --- |
| **Sample** | ***n* = 15** | ***n* = 54** |  |
| Age, year | 12.5 (9-25) | 14.52 ± 5.95 | .944 |
| Height, cm | 159 (142.25-171.5) | 148 ± 18.64 | .100 |
| Weight, kg | 49 (37-78.75) | 49.01 ± 16 | .557 |
| LVEF, % | 58.2 (54.8-60.91) | 51.4 ± 9.95 | .003 |
| LVEDV/BSA, mL/m^2^ | 75.29 (68.47-80.76) | 80.76 ± 21.51 | .867 |
| LVESV/BSA, mL/m^2^ | 30.87 (26.2-33.63) | 40.44 ± 17.92 | .083 |
| SV/BSA, mL/m^2^ | 43.74 (36.86-53.33) | 39.47 (33.69-49.1) | .142 |
| MyoMass/BSA_diast, g/m^2^ | 42.64 (36.8-51.26) | 44.69 (36.76-51.87) | .769 |
| MyoMass/BSA_syst, g/m^2^ | 46.98 (34.76-53.73) | 46.9 (38.51-52.8) | .978 |
| **Strain** |  |  |  |
| RS_Basal | 51.08 (43.98-65.29) | 51.98 ± 22.11 | .334 |
| RS_Mid | 31.07 (26.01-33.94) | 26.87 ± 17.36 | .016 |
| RS_Apical | 28.82 (22.44-45.98) | 27.29 ± 19.91 | .111 |
| RS_Global | 34.72 (27.12-48.24) | 30.91 ± 13.21 | .071 |
| CS_Basal | -17.85 (-19.45--16.6) | -15.58 (-17.14--13.36) | .001 |
| CS_Mid | -18.72 (-19.74--17.5) | -16.05 ± 3.39 | .001 |
| CS_Apical | -20.84 (-21.76--17.79) | -17.51 ± 5.08 | .045 |
| CS_Global | -18.47 (-19.96--17.69) | -16.12 ± 3.19 | .001 |
| LS_Basal | -13.32 (-16.57--9.51) | -9.62 ± 8.54 | .111 |
| LS_Mid | -15.03 (-17.08--12.04) | -11.98 (-14.53--10.25) | .008 |
| LS_Apical | -16.33 (-17.59--14.28) | -14.73 ± 3.25 | .069 |
| LS_Global | -14 (-16.29--11.73) | -12.53 ± 3.31 | .112 |
| **T1 mapping** |  |  |  |
| T1map_seg1 | 1264.98 (1249.66-1336.44) | 1340.31 ± 133.6 | .071 |
| T1map_seg2 | 1267.45 (1232.46-1305.96) | 1308.5 (1275.18-1368.65) | .020 |
| T1map_seg3 | 1286.74 (1222.25-1317.69) | 1294.65 (1260.13-1342.44) | .184 |
| T1map_seg4 | 1261.53 (1222.34-1309.45) | 1306.8 ± 72.47 | .078 |
| T1map_seg5 | 1241.51 (1198.09-1302.61) | 1309.1 ± 102.3 | .003 |
| T1map_seg6 | 1243.31 (1209.05-1296.29) | 1315.89 ± 79.02 | .004 |
| T1map_seg7 | 1280.93 (1234.3-1360.51) | 1337.33 ± 117.71 | .162 |
| T1map_seg8 | 1276.33 (1232.7-1326.76) | 1312.98 (1271.59-1345.21) | .057 |
| T1map_seg9 | 1267.22 (1235.89-1330.24) | 1294.35 (1267.83-1349.23) | .105 |
| T1map_seg10 | 1262.96 (1220.97-1338.48) | 1290.05 (1247.37-1341.52) | .282 |
| T1map_seg11 | 1233.24 (1210.89-1309.62) | 1327.56 (1271.37-1380.09) | .001 |
| T1map_seg12 | 1289.15 (1233.01-1367.75) | 1343.03 ± 94.63 | .158 |
| T1map_seg13 | 1302.56 (1247.02-1407.64) | 1383.86 ± 109.68 | .023 |
| T1map_seg14 | 1307.07 (1222.69-1399.42) | 1370.06 ± 92.22 | .054 |
| T1map_seg15 | 1285.1 (1241.03-1377.23) | 1354.21 ± 105.35 | .061 |
| T1map_seg16 | 1307.13 (1232.66-1397) | 1385.4 ± 88.07 | .012 |

Data are presented as mean ± SD for normally distributed variables and as median (interquartile range, IQR) for non-normally distributed variables. Categorical variables are presented as *n* (%). Between-group comparisons were performed using the Mann–Whitney *U* test for non-normally distributed continuous variables. Categorical variables were compared using Fisher’s exact test. Segmental native T1 values correspond to the AHA 17-segment.

AHA, American Heart Association; BSA, body surface area; LVEDV, left ventricular end-diastolic volume; LVEF, left ventricular ejection fraction; LVESV, left ventricular end-systolic volume; MyoMass, myocardial mass; RS/CS/LS, radial/circumferential/longitudinal strain; SD, standard deviation; SV, stroke volume; T1map, native T1 mapping (ms).

# Table S4. Pairwise comparison of AUCs for discrimination of Group A versus Group B (DeLong test).

| **Comparison** | **Z** | ***P* value** | **ΔAUC** | **SE(ΔAUC)** | **95% CI** |
| --- | --- | --- | --- | --- | --- |
| LVEF vs CS_Mid | -0.315 | 0.753 | -0.014 | 0.330 | -0.104, 0.075 |
| LVEF vs CS_Mid  + T1map_seg11 | -1.218 | 0.223 | -0.060 | 0.321 | -0.157, 0.037 |
| LVEF vs T1map_seg11 | 1.138 | 0.255 | 0.113 | 0.377 | -0.082, 0.308 |
| CS_Mid vs CS_Mid  + T1map_seg11 | -1.150 | 0.250 | -0.046 | 0.315 | -0.124, 0.032 |
| CS_Mid vs T1map_seg11 | 1.237 | 0.216 | 0.127 | 0.374 | -0.074, 0.329 |
| CS_Mid + T1map_seg11 vs T1map_seg11 | 2.354 | 0.019 | 0.173 | 0.359 | 0.029, 0.317 |

Pairwise comparisons of AUCs were performed using the DeLong test. Two-sided p-values are reported and rounded to three decimal places.

Abbreviations: AUC, area under the curve; CS, circumferential strain; LGE, late gadolinium enhancement; LVEF, left ventricular ejection fraction; ROC, receiver operating characteristic; vs, versus.

**Table S5. Firth Penalized Regression Results between Group C and Group D.**

| **Parameters** | **β** | ***P* value** | **aOR, Exp(B)** |
| --- | --- | --- | --- |
| Age | 0.145 | .154 | 1.157 |
| T1map_seg5 | 0.024 | .080 | 1.024 |
| CS_Global | 1.564 | .012 | 4.779 |
| Constant | -10.264 | .506 | — |

# Table S6. Pairwise comparison of AUCs for age-adjusted for discrimination of Group C versus Group D (DeLong test).

| **Comparison** | **Z** | **P value** | **ΔAUC** | **SE(ΔAUC)** | **95% CI for ΔAUC** |
| --- | --- | --- | --- | --- | --- |
| Age vs Age + CS_Global | -2.126 | 0.034 | -0.155 | 0.320 | -0.298, -0.012 |
| Age vs Age + T1map_seg5 | -1.050 | 0.294 | -0.065 | 0.347 | -0.187, 0.057 |
| Age vs Age + CS_Global + T1map_seg5 | -2.194 | 0.028 | -0.160 | 0.312 | -0.304, -0.017 |
| Age + CS_Global vs Age + T1map_seg5 | 1.444 | 0.149 | 0.090 | 0.297 | -0.032, 0.211 |
| Age + CS_Global vs Age + CS_Global + T1map_seg5 | -0.572 | 0.567 | -0.005 | 0.245 | -0.024, 0.013 |
| Age + T1map_seg5 vs Age + CS_Global + T1map_seg5 | -1.642 | 0.101 | -0.095 | 0.287 | -0.209, 0.018 |

Pairwise comparisons of ROC AUCs were performed using the DeLong test. Two-sided p-values are reported and rounded to three decimal places.

Abbreviations: AUC, area under the curve; CS, circumferential strain; ROC, receiver operating characteristic; T1map, vs, versus.

# Table S7. Non-significant baseline and CMR parameters in controls, Group A, and Group B

| **Demographic profile** | **Controls** | **Group A** | **Group B** |
| --- | --- | --- | --- |
| **sample** | **N=30** | **N=16** | **N=39** |
| Height, cm | 156.43±18.72 | 140.5 (134.25-150.75) * | 150.9±19.25 |
| Weight, kg | 53.5 (35.25-71.25) | 40.5 (35.75-50.25) | 51.91±16.78 |
| **T1mapping** |  |  |  |
| T1map_seg1 | 1280.55 (1251.73-1350.14) | 1293.11 (1245.66-1470.75) | 1291.44 (1254.27-1384.87) |
| T1map_seg2 | 1295.38 (1252.51-1318.44) | 1332.01 (1246.67-1386.54) | 1297.14 (1274.95-1363.62) |
| T1map_seg3 | 1297.41±87.51 | 1304.54 (1240.83-1380.78) | 1290.80±54.62 |
| T1map_seg4 | 1285.95 (1233.25-1316.45) | 1270.96 (1238.31-1367.26) | 1294.93 (1237.02-1360.65) |
| T1map_seg7 | 1283.81 (1246.26-1379.31) | 1283.96 (1266.49-1307.43) | 1315.78 (1263.08-1382.46) |
| T1map_seg8 | 1306.75 (1257.9-1330.42) | 1297.41 (1263.68-1333.73) | 1308.72 (1260.75-1350.53) |
| T1map_seg9 | 1272.89 (1247.85-1331.44) | 1293.29 (1254.03-1336.29) | 1310.23±60.43 |
| T1map_seg10 | 1265.34 (1225.32-1317.79) | 1254.88 (1215.25-1305.54) | 1290.65 (1251.35-1353.45) |
| T1map_seg12 | 1304.53±74.5 | 1271.24 (1236.74-1346.96) * | 1326.66 (1272.91-1451.69) |
| T1map_seg13 | 1355.36±101.1 | 1299.01 (1237.47-1422.82) | 1377.81±112.08 |
| T1map_seg14 | 1349.89 (1253.66-1438.75) | 1324.42 (1288.67-1398.84) | 1336.01 (1285.26-1404.1) |
| T1map_seg15 | 1325.22±110.88 | 1273.26 (1250.21-1339.47) | 1350.51±96.73 |
| T1map_seg16 | 1360.74 (1273.41-1451.93) | 1299.92 (1262.28-1465.04) | 1374.5 (1303.88-1422.98) |

Data are presented as mean ± SD for normally distributed variables and as median (interquartile range, IQR) for non-normally distributed variables (distribution assessed before analysis). Between-group comparisons were performed using the Kruskal–Wallis test for non-normally distributed continuous variables. Post hoc pairwise comparisons were adjusted using the Bonferroni method.

BSA, body surface area; SV, stroke volume; T1map, native T1 mapping (ms). Segmental native T1 values correspond to the AHA 17-segment. * p < 0.05 vs controls. Abbreviations: SV, stroke volume; BSA, body surface area.

# Table S8. Non-significant CMR parameters in controls, GroupA, Group C, and Group D.

| **T1mapping** | **Controls** | **Group A** | **Group C** | **Group D** |
| --- | --- | --- | --- | --- |
|  | **N=30** | **N=16** | **N=23** | **N=16** |
| T1map_seg1 | 1280.55 (1251.73-1350.14) | 1293.11 (1245.66-1470.75) | 1299.38 (1270.8-1427.24) | 1284.88 (1215.86-1347.7) |
| T1map_seg2 | 1295.38 (1252.51-1318.44) | 1332.01 (1246.67-1386.54) | 1296.63 (1275.26-1364.5) | 1299.14 (1238-1336.31) |
| T1map_seg3 | 1297.41±87.51 | 1304.54 (1240.83-1380.78) | 1294.16 (1262.07-1329.91) | 1280.77 (1222.49-1305.09) |
| T1map_seg4 | 1285.95 (1233.25-1316.45) | 1270.96 (1238.31-1367.26) | 1267.26 (1237.02-1360.65) | 1308.5 (1245.72-1359.89) |
| T1map_seg6 | 1264.08 (1213.66-1331.49) | 1255 (1219.04-1376.65) | 1331.64 (1254.47-1407.12) | 1321.87 (1286.03-1376.26) |
| T1map_seg7 | 1283.81 (1246.26-1379.31) | 1283.96 (1266.49-1307.43) | 1323.08 (1263.9-1410.11) | 1309.32 (1251.4-1379.42) |
| T1map_seg8 | 1306.75 (1257.9-1330.42) | 1297.41 (1263.68-1333.73) | 1312.67 (1260.75-1350.53) | 1301.83 (1255.78-1349.86) |
| T1map_seg9 | 1272.89 (1247.85-1331.44) | 1293.29 (1254.03-1336.29) | 1293.71 (1251.65-1360.01) | 1297.89 (1269.3-1340.34) |
| T1map_seg10 | 1265.34 (1225.32-1317.79) | 1254.88 (1215.25-1305.54) | 1286.83 (1247.41-1336.05) | 1330.69 (1272.56-1376.1) |
| T1map_seg12 | 1304.53±74.5 | 1271.24 (1236.74-1346.96) | 1310.75 (1270.96-1459.27) | 1345.47 (1289.51-1437.42) |
| T1map_seg13 | 1355.36±101.1 | 1299.01 (1237.47-1422.82) | 1373.45 (1308.93-1445.87) | 1351.08 (1331.43-1399.51) |
| T1map_seg14 | 1349.89 (1253.66-1438.75) | 1324.42 (1288.67-1398.84) | 1331.99 (1270.18-1404.1) | 1346.71 (1296.85-1426.19) |
| T1map_seg15 | 1325.22±110.88 | 1273.26 (1250.21-1339.47) | 1312.78 (1271.3-1379.66) | 1343.73 (1295.19-1446.78) |
| T1map_seg16 | 1360.74 (1273.41-1451.93) | 1299.92 (1262.28-1465.04) | 1376.6 (1297.86-1422.98) | 1363.7 (1329.48-1419.2) |

Data are presented as mean ± SD for normally distributed variables and as median (interquartile range, IQR) for non-normally distributed variables (distribution assessed before analysis). Between-group comparisons were performed using the Kruskal–Wallis test for non-normally distributed continuous variables. Post hoc pairwise comparisons were adjusted using the Bonferroni method.

Fig.S1. Major differential CMR parameters across controls, Group A, and Group B. (A) LVEF, (B) LVEDV/BSA, (C) LVESV/BSA, (D) MyoMass/BSA (diastolic), (E) MyoMass/BSA (systolic), (F) RS_Mid, (G) CS_Basal, (H) CS_Apical, (I) CS_Global, (J) T1map_seg5. ns, not significant; *p < 0.05, **p < 0.01, ***p < 0.001, ****p < 0.0001.

Fig.S2. Major differential CMR parameters across controls, Group C, and Group D. (A) LVEF; (B) CS at basal level; (C) CS at mid-level; (D-G) RS at global, basal, mid, and apical levels; (H) CS at apical level; (K) LS at apical level. ns = not significant, *P<0.05, **P<0.01, ***P<0.001, ****P<0.0001.
